# Microbial Ecology of French Dry Fermented Sausages and Mycotoxin Risk Evaluation During Storage

**DOI:** 10.3389/fmicb.2021.737140

**Published:** 2021-11-04

**Authors:** Monika Coton, Franck Deniel, Jérôme Mounier, Rozenn Joubrel, Emeline Robieu, Audrey Pawtowski, Sabine Jeuge, Bernard Taminiau, Georges Daube, Emmanuel Coton, Bastien Frémaux

**Affiliations:** ^1^Laboratoire Universitaire de Biodiversité et Ecologie Microbienne, Univ Brest, Plouzané, France; ^2^IFIP French Pork Research Institute, Maisons-Alfort, France; ^3^Faculté de Médecine Vétérinaire, Laboratoire de Microbiologie des Denrées Alimentaires, Fundamental and Applied Research for Animal and Health (FARAH), Université de Liège, Liège, Belgium

**Keywords:** fermented sausages, microbial ecology, metabarcoding, fungi, mycotoxins

## Abstract

Dry fermented sausages are produced worldwide by well-controlled fermentation processes involving complex microbiota including many bacterial and fungal species with key technological roles. However, to date, fungal diversity on sausage casings during storage has not been fully described. In this context, we studied the microbial communities from dry fermented sausages naturally colonized or voluntarily surface inoculated with molds during storage using both culture-dependent and metabarcoding methods. Staphylococci and lactic acid bacteria largely dominated in samples, although some halotolerant genera (e.g., *Halomonas*, *Tetragenococcus*, and *Celerinatantimonas* spp.) were also frequently observed. Fungal populations varied from 7.2 to 9.8 log TFU/cm^2^ sausage casing during storage, suggesting relatively low count variability among products. Fungal diversity identified on voluntarily inoculated casings was lower (dominated by *Penicillium nalgiovense* and *Debaryomyces hansenii*) than naturally environment-inoculated fermented sausages (colonized by *P. nalgiovense*, *Penicillium nordicum*, and other *Penicillium* spp. and sporadically by *Scopulariopsis* sp., *D. hansenii*, and *Candida zeylanoïdes*). *P. nalgiovense* and *D. hansenii* were systematically identified, highlighting their key technological role. The mycotoxin risk was then evaluated, and *in situ* mycotoxin production of selected mold isolates was determined during pilot-scale sausage productions. Among the identified fungal species, *P. nalgiovense* was confirmed not to produce mycotoxins. However, some *P. nordicum*, *Penicillium chrysogenum*, *Penicillium bialowienzense*, *Penicillium brevicompactum*, and *Penicillium citreonigrum* isolates produced one or more mycotoxins *in vitro. P. nordicum* also produced ochratoxin A during pilot-scale sausage productions using “worst-case” conditions in the absence of biotic competition. These data provide new knowledge on fermented sausage microbiota and the potential mycotoxin risk during storage.

## Introduction

Dry fermented sausages are among one of the main meat-based traditional fermented foods largely produced and consumed not only in Europe, especially Mediterranean regions, but also worldwide. Among them, a few well-known examples include Saucisson sec or Rosette in France, Salami in Italy, or Fuet in Spain. In France, nearly 113,000 tons of dry-cured sausages were produced in 2019, which actually represents about 10% of the global processed meat production^1^. On average, 75,000 tons are consumed yearly in France, and many dry fermented sausages are exported worldwide. When it comes to product export, scientific data as well as methods to determine overall product quality and safety are required. In the case of dry-cured sausages, knowledge about the fungal species encountered not only during ripening and drying but also during storage, which are either inoculated onto the sausage casing or are part of the in-house mycobiota (air, equipment, drying rooms, etc.), and their potential to produce mycotoxins is needed.

Mycotoxins are secondary metabolites produced by different fungal species that can be potentially toxic for consumers following ingestion of contaminated foodstuffs. The deleterious health effects of these mycotoxins (neurotoxicity, hepatoxicity, cancerogenicity, or nephrotoxicity) can appear after acute, short-term, and/or long-term exposures (ANSES, 2009; Hymery et al., 2014). In the case of some foods, mycotoxins may be found on the surface and/or potentially migrate into the food matrix depending on whether the fungal strain present is actually mycotoxigenic but also on product composition [water activity (*a*_*w*_), pH, etc.] and storage conditions (temperature, duration, etc.) (Coton and Dantigny, 2019). The European regulation (EC) N° 1881/2006 set maximum limits for some mycotoxins in certain food categories prone to mold contamination but not fermented meat (Official Journal of the European Union, 2006). However, according to the considered country, similar criteria also exist for food import and/or export including some fermented foods (breads) or beverages (wine and cider).

Mycotoxins are especially well known to be produced by species belonging to the *Fusarium*, *Aspergillus*, and *Penicillium* genera. In particular, dry spores (xerospores) of *Aspergillus* and *Penicillium* genera are most often encountered among the complex microbial communities found on the surface of fermented meats such as dry-cured sausages (Castegnaro and Pfohl-Leszkowicz, 2002; Battilani et al., 2007; Iacumin et al., 2009; Sonjak et al., 2011; Magista‘ et al., 2017; Franciosa et al., 2021), as they resist low *a*_*w*_, low pH, and high salt concentrations. Molds associated with dry-cured sausage casings either come from the in-house mycobiota or are purposely inoculated onto casings by spraying or dipping before the ripening and drying stages. The subsequent fungal growth will directly impact the overall product quality (aromas, flavors, color, protection against pathogens, etc.). Molds can reach high levels on dry-cured sausages ranging from 5 to 8 log TFU/g (F. Deniel, personal communication). The most frequently encountered fungal species of technological interest is *Penicillium nalgiovense* (considered as atoxinogenic), but other species, such as *Penicillium salamii* species (also considered atoxinogenic) or *Penicillium chrysogenum* and *Penicillium nordicum* (both being potentially mycotoxigenic), have been described (Leistner, 1990; Sunesen and Stahnke, 2003; Iacumin et al., 2009; Perrone et al., 2015; Magista et al., 2016; Perrone, 2017; Franciosa et al., 2021). Other fungal species, often coming from the working environment, may colonize sausages and be mycotoxigenic (e.g., *Penicillium olsonii*, *Penicillium expansum*, *Penicillium viridicatum*, *Penicillium granulatum*, *Penicillium oxalicum*, *Penicillium commune*, *Aspergillus versicolor*, or *Aspergillus fischeri*) (Samson et al., 2004, 2010; Sørensen et al., 2008; Iacumin et al., 2009; Canel et al., 2013; Montanha et al., 2018). Species belonging to the *Cladosporium*, *Mucor*, *Scopulariopsis*, or *Geotrichum* genera may also be sporadically encountered (Canel et al., 2013; Magista‘ et al., 2017). Although the development of some fungal species on sausage casing is essential during drying and ripening, the presence of potentially mycotoxigenic contaminating species may pose a mycotoxin risk not only on the sausage surface but also if diffusion occurs into the meat. Among the above-cited species, some have already been described to produce patulin (PAT), ochratoxin A (OTA), citrinin (CIT), or cyclopiazonic acid (CPA) in fermented meats (Wu et al., 1974; Escoula, 1992; Pohland et al., 1992; Tabuc et al., 2004; Dijksterhuis, 2007; Iacumin et al., 2009), although their presence does not systematically mean that mycotoxins will be produced as both abiotic and biotic factors will have an impact. Therefore, the mycotoxin risk needs to be evaluated using fungal species actually encountered in dry fermented sausages and determining their ability to produce these compounds *in situ*. To fill in this gap, we studied the microbial communities of dry fermented French sausages that were either naturally or voluntarily surface inoculated with molds. Sausage casings were then monitored at different time points from ripening to the end of storage using both culture-dependent and -independent methods. *In vitro* mycotoxin production for representative fungal isolates was determined and compared to the values obtained for mycotoxin contents on sausage casings as well as after potential diffusion into the meat. Finally, mycotoxigenic strains were used in pilot-scale fermented sausage productions to determine the *in situ* potential to produce mycotoxins and evaluate the associated mycotoxin risk.

## Materials and Methods

### Sample Preparation and Microbial Numerations

Ten dry fermented sausages, five naturally surface inoculated with in-house mycobiota and five others voluntarily inoculated with commercial mold cultures (all including *P. nalgiovense*) and bacterial starters (potentially including *Latilactobacillus sakei*, *Staphylococcus xylosus*, and *Staphylococcus carnosus* according to producer) during manufacture, were monitored during storage. These products came from nine different producers located in six different departments in France. The five naturally surface-inoculated sausages were all made using natural casings (i.e., large “chaudin” or small “menu” pork intestine casings), varied in weight from 250 to 300 g, and were stored without any protective packaging except in one case (macroperforated packaging used). For sausages voluntarily surface inoculated with commercial molds, they were made using collagen casings (4/5; weights varied from 5 × 75 to 400 g) or natural casing (1/5; 250 g weight) and were stored in protective atmosphere packaging (3/5) or with macroperforated packaging (2/5). Table 1 summarizes the sample characteristics.

**TABLE 1:** Characteristics and conservation conditions of the studied dry fermented sausages (S1–S10).

Code	Casing	Commercial or indigenous molds	Weight (g)	Edible casing (yes/no)	Packaging	Conservation scenario
**S1**	Collagen	C	400	N	Macroperforated	60 days, 18°C
**S2**	Natural chaudin	I	280	Y	None	60 days, 18°C
**S3**	Natural chaudin	C	250	Y	Macroperforated	60 days, 18°C
**S4**	Natural menu	I	300	Y	Macroperforated	60 days, 18°C
**S5**	Natural chaudin	I	300	Y	None	60 days, 18°C
**S6**	Natural chaudin	I	250	Y	None	60 days, 18°C
**S7**	Natural chaudin	I	290	Y	None	120 days, 18°C
**S8**	Collagen	C	5 × 75	Y	Protective atmosphere	120 days, 18°C
**S9**	Collagen	C	200	N	Protective atmosphere	120 days, 18°C
**S10**	Collagen	C	250	N	Protective atmosphere	120 days, 18°C

All samples (both meats and casings) were analyzed at “day 0,” which corresponded to the time when sausages were removed from the drying chamber and packaged, and at the end of shelf life (either 60 or 120 days according to manufacturer recommendations) (Table 1). During conservation, sausages were stored in large rectangular perforated closed containers (volume 1 m^3^), one per producer, at ambient temperature conditions (∼20°C). Each sausage was individually hung on a string to avoid contact between samples and with the container.

For each sampling date and each product, casing and meat samples corresponded to a pool of five sausages from five different production batches. For casings, 5 cm × 1 cm pieces were removed and pooled together. Three biological replicates were then performed from each pool for analyses. For sausage casing samples, physicochemical analyses, fungal and bacterial counts, mycotoxin determinations, and total DNA extractions for metabarcoding analyses were performed. Meat was also sampled and analyzed for physicochemical parameters, bacterial counts (using 10 g fractions), mycotoxin content at two different depths (4 g from the first 0.5 cm depth into the meat as well as 4 g from the 0.5 to 1 cm depth), and metabarcoding analyses.

For microbial counts from casings, samples were first resuspended in sterile Tween (0.01% *v*/*v*) water and then vortexed for 1 min before plating 10-fold serial dilutions onto the surface of different media. Yeast extract glucose chloramphenicol (YGC) (bioMérieux, France) was used to enumerate total yeasts and molds (incubation 25°C for 72–96 h) according to the NF V08-059 (2002) standard, while malt extract agar (M2Lev) (malt extract 20 g/l and yeast extract 3 g/l) supplemented with penicillin (5 mg/l) and streptomycin (5 mg/l) (incubation at 25°C for 1 week) and M5S5 (malt extract 50 g/l and salt 50 g/l), specifically for molds requiring low water activity conditions (incubation 25°C, 7 days), were used to enumerate yeasts and filamentous fungi on casings. De Man, Rogosa, and Sharpe (MRS) (AES, France) was used for lactic acid bacteria (anaerobic incubation for 48–72 h at 30°C) and plate count agar (PCA; according to the ISO 4833-1:2013) (AES, France) for total mesophilic bacteria (72 h at 30°C). For molds, thalli were selected according to their macroscopic and morphological aspects from a representative Petri dish for each sampling date; then, microscopic evaluations were performed to select representative isolates from each microbial group for species-level identifications. For yeasts, the square root of the total number of isolates was used to determine how many isolates were to be conserved.

For microbial counts from meat samples, 10 g was first homogenized with an ULTRA-TURRAX (IKA, Germany) and then placed in a sterile Stomacher bag and mixed for 2 min in 90 ml tryptone salt buffer using a Stomacher (AES, France). Homogenates were serially diluted and plated on PCA (incubation 72 h at 30°C) (AES, France) for total aerobic bacteria, MRS (anaerobic conditions for 72–96 h at 30°C, according to the ISO 15214:1998) (AES, France) for lactic acid bacteria, and YGC (72 h at 25°C) (bioMérieux, France) for yeast and molds. Representative molds and yeasts were cryoconserved at −80°C in a 20% *v*/*v* glycerol solution.

### Physicochemical Analyses

Water activity (*a*_*w*_) and pH values for both casing and meat samples were determined according to the NF ISO 21807:2005 and NF V04–408 standards, respectively. *A*_*w*_ and pH measurements were performed using an AQUALAB 4TEV (Meter, Germany) and a FiveGo (Mettler-Toledo, Switzerland) apparatus connected to a puncture electrode LE427-IP67 (Mettler-Toledo, Switzerland), respectively.

### Yeast Fourier-Transform Infrared Spectroscopy Dereplication and Species Identifications

#### Sample Preparation and Fourier-Transform Infrared Analyses

Sample preparation, measurement, and high-throughput Fourier-transform infrared (FTIR) spectral analyses were performed according to Kümmerle et al. (1998) and Coton et al. (2017) on an FTIR system equipped with a spectrometer (Tensor 27, Bruker Optics, Champs sur Marne, France) coupled to a high-throughput module (HTS-XT, Bruker Optics). Three FTIR technical replicates were performed for each isolate, and dendrograms were created using OPUS software (Bruker, France). Yeast isolates were grouped based on their FTIR spectra, then presumptive identifications were done using the Technical University of Munich reference database consisting of about 2,500 FTIR spectra of type and reference yeast strains.

#### Molecular Identifications of Yeast and Mold Isolates

Based on macroscopic and microscopic observations, representative mold isolates were identified to the species level after sequencing either the partial β-tubulin (using Bt2a-GGTAACCAAATCGGTGCTGCTTTC/Bt2b-ACCCTCAGTG TAGTGACCCTTGGC for *Penicillium* isolates), actin gene (ACT-512f-ATGTGCAAGGCCGGTTTCGC/ACT-783R-TAC GAGTCCTTCTGGCCCAT for *Cladosporium* isolates), or internal transcribed spacer (ITS) region (using ITS5-GGAA GTAAAAGTCGTAACAAGG/ITS4-TCCTCCGCTTATTGAT ATGC for other fungal species). PCR amplifications were carried out as follows: for ITS, using 0.2 μM each primer, 2 mM MgCl_2_, 200 μM dNTPs, 1× buffer, and 0.625 U *Taq* polymerase (GoTaq, Promega, France); for β-tubulin, using 0.2 μM each primer, 1.5 mM MgCl_2_, 200 μM dNTPs, 1× buffer, and 0.625 U *Taq* polymerase (GoTaq, Promega, France); and for actin, using 0.5 μM each primer, 2 mM MgCl_2_, 200 μM dNTPs, 1× buffer, and 1.25 U *Taq*. Amplification conditions were as follows: for β-tubulin, 95°C for 5 min, 35 cycles of 95°C for 60 s, 61°C for 60 s, 72°C for 60 s, then 72°C for 5 min and for actin, 95°C for 8 min, 35 cycles of 95°C for 15 s, 58°C for 20 s, 72°C for 60 s, and then 72°C for 5 min.

For yeasts, amplification and sequencing were performed using the NL-1-GCATATCAATAAGCGGAGGAAAAG and NL-4-GGTCCGTGTTTCAAGACGG primers (Kurtzman and Robnett, 1997) in the presence of 0.2 μM each primer, 1.5 mM MgCl_2_, 0.2 mM dNTPs, 1× buffer, and 0.625 U *Taq* polymerase (GoTaq, Promega, France). Amplification conditions were as follows: 95°C for 2 min, 36 cycles of 95°C for 60 s, 52°C for 90 s, 72°C for 60 s, and then 72°C for 10 min.

All amplifications were performed using a peqSTAR 2× Gradient Thermocycler (PEQLAB Biotechnologie GMBH, Erlangen, Germany). PCR sample aliquots (9 μl) were analyzed using 0.8% (*w*/*v*) agarose gels (Promega, France) in 1× TBE buffer at 110 V for 50 min and then visualized with GelRed staining (Biotium, France). All PCR products were sequenced by Eurofins (Abersberg, Germany), and species-level identifications were done using standard BLASTN searches against the nr database in GenBank.

### Metabarcoding Analyses

#### Total DNA Extractions

DNA extractions were performed in triplicate using kits for total bacterial and fungal DNA (MP Biomedicals, United States) with some minor modifications. Briefly, either 5 g × 4 g meat samples or 5 cm × 1 cm casing samples were analyzed for each sample type and date. The 20-g meat samples were first cut into small pieces, and then 80 ml sterile Tween (0.01% *v*/*v*) water was added before homogenization with an ULTRA-TURRAX for 1 min at 14,000 rpm. Then, 4 ml × 5 ml homogenates were centrifuged at 8,000 *g* for 15 min, and cell pellets were stored at −20°C until use. For the casing samples, 9 ml sterile Tween (0.01% *v*/*v*) water was added and then thoroughly vortexed to release microbial cells from the surface, before removing the casings. The obtained homogenates were centrifuged at 8,000 *g* for 15 min, and cell pellets were stored at −20°C until use. For DNA extractions, slightly thawed cell pellets were resuspended in 500 μl yeast lysis solution, divided in two, and then the protocol was followed as described by the manufacturer. After extraction, DNA samples were purified using the DNeasy Tissue Kit silica-based columns (Qiagen, France) according to the manufacturer’s instructions. Pooled DNA quality and quantity were verified by gel electrophoresis and NanoDrop (LabTech, France), respectively, prior to storage at −20°C.

#### Targeted 16S rRNA Gene and Internal Transcribed Spacer Metabarcoding Analyses

For bacterial communities, the V1–V3 region of the 16S rRNA gene was amplified, and library preparation was performed using the following primers with Illumina overhand adapters: Bact-FOR-TCGTCGGCAGCGTCAGATGTGTATAAGAGACAG and Bact-REV-GTVTVGTGGGCTCGGAGATGTGTATAAGAGA CAG-3′. As for fungal communities, the ITS region was amplified using the ITS3-GCATCGATGAAGAACGCAGC and ITS4_Kyo4-TCCTCCGCTTWTTGWTWTGC primer pair. All PCR products were purified using the Agencourt AMPure XP beads kit (Beckman Coulter, Pasadena, CA, United States) before a second PCR round for indexing, using the Nextera XT index primers 1 and 2. After purification, PCR products were quantified with Quant-iT PicoGreen (Thermo Fisher Scientific, Waltham, MA, United States) and concentrations adjusted to 10 ng/μl. A final quantification, by qPCR, of each sample in the library was performed using the KAPA SYBR FAST qPCR Kit (Kapa Biosystems, Wilmington, MA, United States) before normalization, pooling, and sequencing on a MiSeq sequencer using v3 reagents (Illumina, United States).

#### Bioinformatics Analyses of Metabarcoding Data

The DADA2 library (Callahan et al., 2016) was used in R version 3.5.0 (R Core Team, 2017) for 16S rRNA gene and ITS reads filtering. For 16S rRNA gene reads, forward and reverse read pairs were trimmed and filtered, with forward reads truncated at 290 bp and reverse reads at 270 bp, and at the first quality score of 2, no ambiguous bases were allowed and each read was required to have less than five expected errors based on their quality scores. For ITS reads, the same parameters were applied except that each read was required to have less than two expected errors based on their quality scores. Amplicon sequence variants (ASVs) were independently inferred from the forward and reverse reads of each sample using the run-specific error rates, and then, read pairs were merged requiring at least 15-bp overlap. For ITS reads, prior to chimera filtering, the ITS2 region was extracted using ITSx v1.0.11 (Bengtsson-Palme et al., 2013) and the *-t F* option to only obtain fungal sequences. Then, 16S and ITS chimera sequences were removed using UCHIME algorithm (Edgar et al., 2011) implemented in VSEARCH v1.1.3^2^ against the ChimeraSlayer reference database (Haas et al., 2011) and UCHIME reference dataset version 7.2 (Unite Community, 2017), respectively. The RDP classifier (Wang et al., 2007) was used for 16S rRNA sequence taxonomy assignment, which was made with Greengenes v13.8 database (McDonald et al., 2012) available in QIIME (Caporaso et al., 2010). For ITS2 sequences, BLAST algorithm (Altschul et al., 1990) was used for taxonomic assignment, which was made with UNITE plus INSD non-redundant ITS database version 8.0 (Unite Community, 2019). Home-made ruby scripts were used to correct misunderstood parameters reported by Shah et al. (2019). Briefly, *blastn* analysis was done using 150 as the number of hits selected by the parameter *max_target_seqs*, and then, the first hit in the list, presenting the highest score and percentage of identity, was selected for taxonomic assignment.

Each 16S and ITS ASV assignment was then verified to check for possible multi-affiliation at the species level using the RDP seqmatch^3^ and BLAST^4^ tools, respectively. Multi-affiliated ASV at the species level were only assigned to the genus level.

Alpha and beta diversity analyses were performed using QIIME and the Calypso software tool v8.84 (Zakrzewski et al., 2017) after normalization on the smallest number of reads found in a sample and after total sum normalization of count data combined with square root transformation (Hellinger transformation), respectively.

All biosample raw reads have been deposited at the National Center for Biotechnology Information (NCBI) and are available under the Bioproject ID PRJNA757247^5^.

#### Statistical Analyses

Statistical differences of population abundance between treatment groups were assessed by using a one-way ANOVA, with multi-testing corrections (Benjamini–Hochberg false discovery rate) using the STAMP software (Parks and Beiko, 2010). Statistical paired differences between treatment groups of specific bacterial populations were assessed by two-way ANOVA and Tukey–Kramer *post hoc* tests using PRISM 6 (GraphPad Software). Differences were considered significant for a *p*-value of less than 0.05.

### Mycotoxin Determination in Dry Fermented Sausages

#### Non-selective Extrolite Extractions From Dry-Cured Sausages

Mycotoxin determination was carried out on sausage casing samples at the final storage dates and, in the case of positive results, meat samples were also tested. For sausage casings, 1 g obtained from five batches was collected and cut into small pieces. Then, mycotoxins were extracted from casings using a 15-ml acetonitrile/methanol/water (ACN/MeOH/H_2_O, 30:30:40 *v*/*v*/*v*) mixture. Samples were first vortexed for 1 min, casings scraped to remove mycelium, and then vortexed 1 min further before incubating for 1 h at room temperature with regular shaking. Samples were then sonicated for 30 min before adding 15 ml hexane.

For meat, 4 g of samples from the uppermost 0.5 cm (obtained from the same five sausage batches as above) was cut into small pieces using a scalpel. Then, 13 ml ACN/MeOH/H_2_O (30:30:40) was added, and the mixture was homogenized for ∼1 min with an ULTRA-TURRAX (IKA, Germany) before adding 12 ml more of ACN/MeOH/H_2_O (same ratios). Samples were then vortexed for 1 min before 30-min sonication, and hexane (20 ml) was added.

All samples (casings or meats) were placed on a Rotoflex for 10 min and then centrifuged at 7,000 *g* for 10 min at 4°C. The ACN/MeOH/H_2_O phase was recovered and stored in amber vials at −20°C until use. Before quadrupole time-of-flight mass spectrometry (Q-TOF LC/MS) analyses, samples were filtered through a 0.45-μm PTFE membrane syringe 4-mm filter (GE Healthcare Life Sciences, Little Chalfont, United Kingdom) into an amber vial.

#### Chromatographic and Mass Spectrometry Conditions

Extracted extrolites were identified by an Agilent 6530 Accurate-Mass Q-TOF LC/MS system (Agilent Technologies, Santa Clara, CA, United States). The LC system included a binary pump 1260 and degasser, a well-plate autosampler with thermostat (set to 10°C), and a thermostated column compartment (set to 35°C). Two microliters of filtered samples were injected into the system equipped with a ZORBAX Extend-C18 column (2.1 mm × 50 mm and 1.8 μm, 600 bar). The flow rate was set to 0.3 ml/min using as mobile phase the following: solvent A (Milli-Q water + 0.1% LC/MS formic acid *v*/*v*) (Carlo Erba Reagents, France) + 0.1% ammonium formate *v*/*v* (Thermo Fisher Scientific, Waltham, MA, United States), and solvent B (LC-MS-grade ACN supplemented with 0.1% formic acid). Solvent B was maintained at 10% for the first 3 min, followed by a gradient of 10–100% of B up to 45 min. Finally, solvent B was maintained at 100% for 5 min followed by 5-min post-time to regenerate the column and come back to initial conditions. Compounds were detected using an Agilent 6530 Series Accurate-Mass Q-TOF. Analytes were ionized in electrospray ionization positive (ESI+) and ESI− mode. Mass spectrometry conditions were as follows: capillary voltage, 4 kV; source temperature, 325°C; nebulizer pressure, 50 psig; drying gas, 12 l/min; and ion range, 100 to 1,000 m/z. Targeted metabolites were aflatoxin B1 (AFB1), andrastin A (ANDA), citreoviridin (CTV), CIT, CPA, eremefortins A and B (EREM A and EREM B, respectively), griseofulvin (GSF), (ISO)-fumigaclavin [(ISO)FUM], meleagrin (MEL), mycophenolic acid (MPA), OTA, PAT, penitrem A (PENA), roquefortine C (ROQC), and sterigmatocystin (ST); compound characteristics are given in Supplementary Table 1. Standards were obtained from Sigma-Aldrich (St. Louis, MO, United States). Extrolite stock solutions (ranging from 1 to 10 mg/ml) were prepared in dimethyl sulfoxide (DMSO) and stored at −20°C in amber vials until use. Linear ranges were prepared in ACN and compared to the matrix-matched linear range to observe any matrix effects.

#### Method Performance and Validation Parameters

Method performance and validation parameters [i.e., linearity (*R*^2^), detection limit (DL), quantification limit (QL), recovery, and matrix effects] were evaluated as described by the ICH guidelines (ICH Harmonized Tripartite Guideline, 2005). For recovery experiments, meat and casing samples were spiked with a mixture of 12 extrolites at 1,000 ng/ml, and recovery percentages were evaluated in triplicate. For each extrolite, the method matrix effect was estimated, and values ranged from 74 to 96% for all except CIT (56% for meat) and STERI (63% for meat) (Supplementary Table 1). A matrix-matched calibration method was also used for reliable extrolite quantification as previously described (Fontaine et al., 2015). To do so, blank matrix extracts from meat samples were obtained using the same extraction procedure as above. The extract was then supplemented with adequate volumes of each target extrolite to obtain concentrations ranging from 5 to 10,000 ng/μl for all extrolites except PAT that ranged from 75 to 150,000 ng/μl. Calibration curves were obtained by plotting the peak area against extrolite concentrations using a 1/*x*^2^ weighted linear regression model. DL and QL values for each extrolite were determined by multiplying the standard deviation of *y*-intercepts of regression lines divided by the slope, by 3.3 and 10, respectively. Calibration curve calculations were carried out using the Agilent MassHunter Workstation Software (Agilent Technologies, Santa Clara, CA, United States).

### *In situ* Mycotoxin Determination in Pilot-Scale Dry Sausage Challenge Tests

#### Manufacture of Pilot-Scale Dry Sausages

For these experiments, two batches of dry fermented sausages were produced in a pilot-scale unit (IFIP, France). Briefly, each batch was prepared with lean pork (80%) and pork back fat (20%) that were ground through a 6-mm plate of a DRC 98 mincer (PSV, France). Afterward, NaCl (26 g/kg), NaNO_2_ (120 mg/kg), KNO_3_ (120 mg/kg), lactose (10 g/kg), dextrose (5 g/kg), ground black pepper (1.5 g/kg), and the commercial starter culture Flora Italia (Chr Hansen, Denmark, following manufacturer’s recommendation) were added to the mixture. The mixture was then divided into two parts, the first one was stuffed into 45–50-mm-diameter natural casings (NC) (Soussana, France) and the second one into 50-mm-diameter collagen casings (CC) (Soussana, France) for comparison. All fresh sausages weighed from 280 to 300 g. Sausages from the first batch were sprayed with a suspension containing equivalent concentrations of two *P. nordicum* isolates (F9M9 and D7M3) selected based on their mycotoxin profiles during this study. Those from the second batch were sprayed with a suspension containing equivalent concentrations of two atoxigenic *P. nalgiovense* isolates (FC35M1 and D5M8) (control batch). Sausages were placed in a temperature- and humidity-controlled incubator for 30 days using a protocol classically used in the meat processing industry (Supplementary Table 4). Finally, they were stored unpacked at 18°C with a relative humidity of 50–60% for up to 100 days for mycotoxin analysis; all other analyses were done at 70 days.

#### Microbiological Analyses of Pilot-Scale Dry Sausages

For each batch (*P. nordicum* vs. *P. nalgiovense*) and casing type (NC vs. CC), dry fermented sausage samples were analyzed for total aerobic mesophilic and lactic acid microbiota at five sampling dates, including D0, D3 (end of ripening step), D15, D30 (end of drying step), and D50 (after 20 days of storage). Total aerobic mesophilic microbiota and lactic acid bacteria (LAB) were enumerated in triplicate (from three different dry fermented sausages) as already described (cf. section “Sample Preparation and Microbial Numerations”). The quantification limit of the microbiological analysis was 10 CFU/g.

Total fungal populations were monitored at D0 (before spraying with the appropriate mold suspension), D0 (after spraying with the appropriate mold suspension), D15, D30, D50, D85, and D100 using the same method described previously (cf. section “Sample Preparation and Microbial Numerations”).

#### Physicochemical Analyses of Pilot-Scale Dry Sausages

For each sausage batch (*P. nordicum* vs. *P. nalgiovense*), relative humidity and temperature during ripening and the drying processes were continuously monitored using a humidity and temperature probe HMP110 (Vaisala, Finland) placed in the chamber atmosphere and connected to a real-time monitoring system Labguard 3D (bioMérieux, France).

For each batch (*P. nordicum* vs. *P. nalgiovense*) and type of casing (NC vs. CC), pH and *a*_*w*_ measurements were performed as described above (cf. section “Physicochemical Analyses”) on meat samples collected in the middle and at one end of three different sausages at D0, D3, D20, D30, and D50. Weight loss (difference between the initial weight and the weight measured during ripening and drying processes) of NC and CC sausages was regularly checked (once a day) during the whole process.

## Results

### Physicochemical Follow-Up of Dry Fermented Sausages During Storage

Conservation scenarios varied from 60 to 120 days with all samples being stored at ambient temperature (Table 1). At both the beginning and end of storage, changes in the physicochemical characteristics were determined for fermented dry sausages naturally covered (*n* = 5) or voluntarily surface inoculated (*n* = 5) with molds (Supplementary Table 2). At the start of conservation, pH values obtained for meat samples were variable and ranged from 4.87 ± 0.12 (average of five sausages/analysis) to 5.77 ± 0.16 and mostly increased by the end of conservation. pH was also determined on the casing (meat–casing interface) and was systematically higher, by up to ∼1 pH unit at the start of conservation (as observed for sausage S1), than in the core. In general, an increase of 0.1 to 1.5 pH units (for both meat and casing samples) was observed during conservation, with the highest increases (>0.5 pH units) observed for S10, S4, S1, S2, and S3.

Water activity (*a*_*w*_) in core samples was also variable and ranged from 0.761 ± 0.04 (for sample S8) to 0.902 ± 0.03 (for sample S2) at the start of the conservation period before decreasing over time as expected. Overall, *a*_*w*_ values decreased for all meat samples by up to 0.15 units, the highest changes being observed for S2, S5, and S6. Surface values were typically lower than core *a*_*w*_ values regardless of the type of casing, and this trend was observed to the end of storage.

### Microbial Dynamics During Dry-Cured Sausage Storage

A dynamic follow-up of bacterial and fungal populations was also done at the start and end of the storage period for the same 10 dry-cured sausages on both sausage casing and meat (core) samples.

At the start of conservation, total aerobic microbial (TAM) counts were high on the 10 casings and ranged from 6.9 (S10) to 9.1 log CFU/g (S4) (Supplementary Table 2). Casing type did not appear to have an impact on these counts, and based on the visual aspects of the colonies and the results for fungal counts (Supplementary Figure 1), they were clearly dominated by fungal isolates that naturally colonized the surface or were intentionally inoculated onto the sausage surface. During storage, TAM counts remained relatively stable or slightly increased for five samples, while they decreased by 0.8–4.2 log CFU/g in the other five sausages, but no link to either casing type or storage time could be established. For meat samples, counts were also high and reached up to 9.2 log CFU/g, before decreasing during storage by ∼1–2 log CFU/g. These counts were observed to be dominated by lactic acid bacteria that were enumerated at very similar counts at the start of storage before decreasing by 1–3 log CFU/g by the end of storage (Supplementary Table 2).

Total fungal populations (yeasts and molds) were high from the start of storage and ranged from 7.2 to 9.8 log TFU/cm^2^ casing; populations also remained relatively stable to the end of storage for all samples (Supplementary Figure 1). Yeast and mold population dynamics were also assessed individually, and differences in yeast-to-mold ratios were observed among samples (Supplementary Figure 1). These differences were mainly due to changes in yeast population dynamics as mold counts remained relatively stable for seven sausages (<1 log TFU/cm^2^ casing) during conservation, and only a ∼1 log TFU/cm^2^ difference was observed for the other three during storage.

### Fungal Isolate Identifications and Phenotypic Diversity of the Main Encountered Species

**FIGURE 1:**
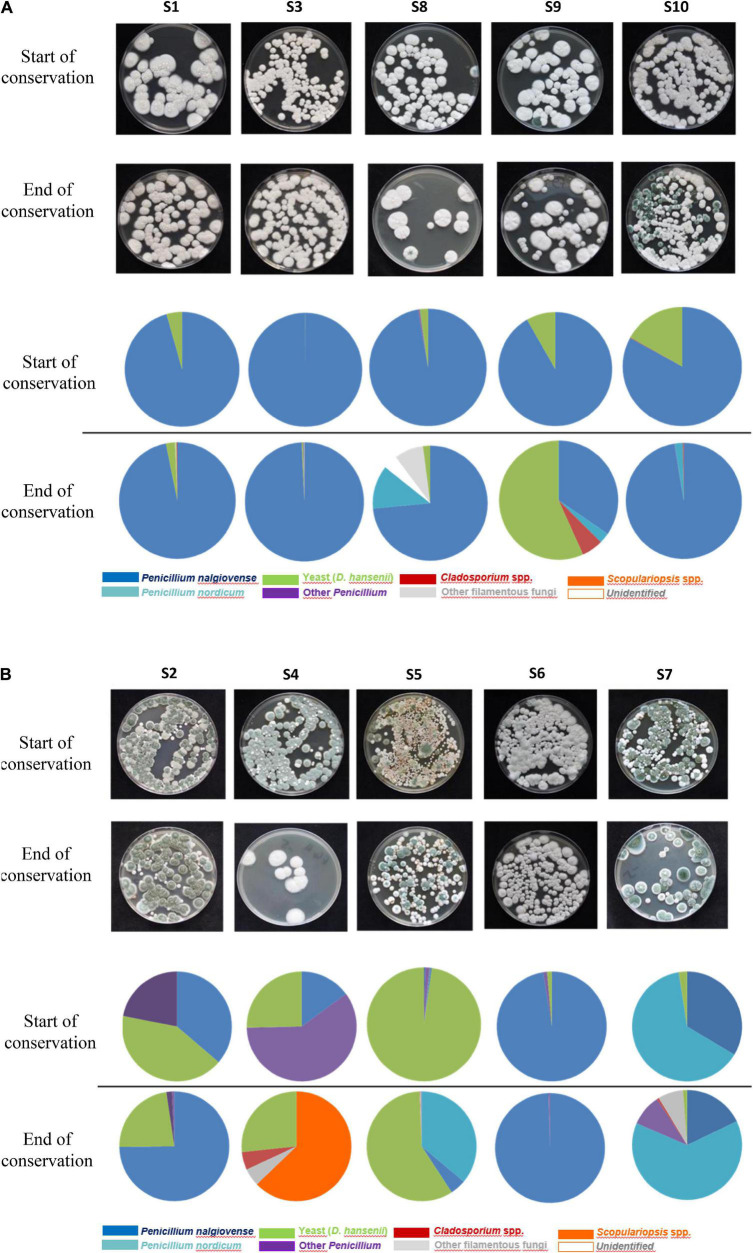
**(A)** Fungal diversity observed on at the start and end of conservation for the five dry fermented sausages voluntarily surface-inoculated with molds. **(B)** Fungal diversity observed at the start and end of conservation for the five dry fermented sausages naturally surface-inoculated with molds.

For all five sausages produced with commercial mold cultures (all containing *P. nalgiovense*) (S1, S3, S8, S9, and S10), *P. nalgiovense* was highly dominant at the start of storage (Figure 1A) as this species was used as the ripening culture during production. This species abundance was identified at >85% on sausage casings for these five sausages. *D. hansenii* was the next most abundant species, another technologically important species often inoculated during production, and was identified in all voluntarily inoculated samples between 1 and 20% (Figure 1A). Both species can be considered to be part of the core microbiota during fermentation. At the end of storage, an increase in fungal species diversity was observed. Other genera and species were identified including *P. nordicum* (identified between 2 and 63% in 3/5 sausages); *Cladosporium* spp. (identified between 1 and 6% in 3/5 sausages); and to a lesser extent other *Penicillium*, *Scopulariopsis*, or *Aspergillus* spp. although their abundances were typically lower than those of technological species.

In comparison to sausages sprayed with commercial fungal suspensions, a higher diversity was observed for the fermented sausages naturally colonized by in-house mycobiota. This diversity was observed from the start of storage and continued to increase over time, except for S6 for which a similar trend to inoculated sausages was observed (Figure 1B). *P. nalgiovense* and *D. hansenii* species were identified in all sausages but at much more variable abundances, and they were not systematically the most abundant species contrary to what was observed for voluntarily inoculated fermented sausages. *P. nordicum* was identified among the most dominant species in S5 and S7 and represented 36 and 63% species abundance, respectively, at the end of storage. This species was also identified in three other sausages but at 2, 3, and 12% abundances. Other predominant species identified in these samples corresponded to *Scopulariopsis* sp., *Cladosporium* sp., or other *Penicillium* spp. such as *P. chrysogenum*.

Among *Penicillium* isolates belonging to the three main identified species during this study, *P. nalgiovense* (*n* = 48), *P. nordicum* (*n* = 14), and *P. chrysogenum* (*n* = 3), the Pitt method was performed to evaluate their morphological diversity. Among *P. nalgiovense* isolates, 16 morphotypes were observed that mainly varied in color due to conidia (from white to green-blue-gray colonies with white borders). They included four main phenotypes that were systematically observed for isolates coming from all 10 fermented sausages (Supplementary Figure 2A). White phenotypes were observed in products voluntarily inoculated with commercial cultures and were logically identical to the isolated commercial strains that we included as references, while green colonies corresponded to wild strains identified on naturally colonized sausages. For *P. nordicum*, three different phenotypes were observed (Supplementary Figure 2B) that also varied in color from white to green, while only two different phenotypes were observed for *P. chrysogenum* isolates and mainly varied in color from pale green to darker green with white borders (Supplementary Figure 2C).

### Microbial Community Diversity and Dynamics During Dry-Cured Sausage Fermentations Determined by Metabarcoding Analyses

**FIGURE 2:**
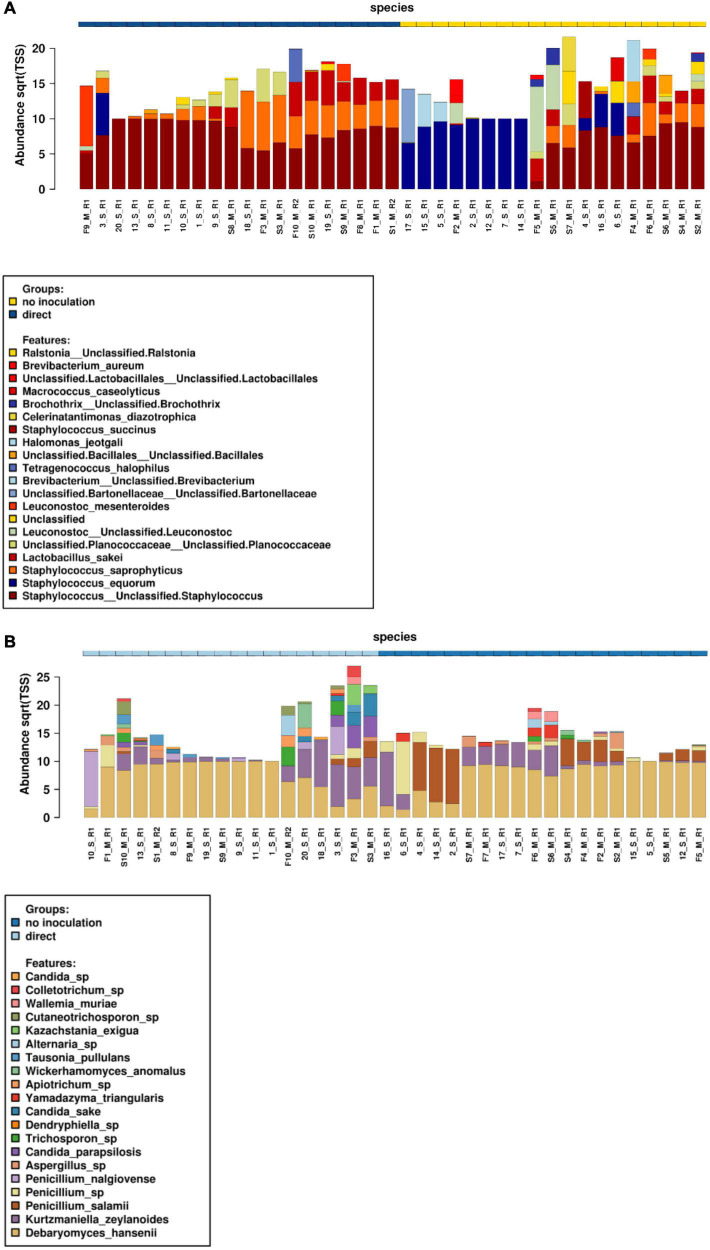
Relative abundances of bacterial **(A)** and fungal **(B)** communities at the start and end of conservation for both meat and casing samples. S, start date meat samples (indicated as S1–S10); F, finish date meat samples (indicated as F1 to F10); 1–10, start date for surface casing samples; 11–20, finish date for surface casing samples; M, meat; S, sausage casing; R, replicate.

Metabarcoding high-throughput sequencing was performed on both sausage casing and meat samples at both the start and end of storage for all 10 sausages. Relative abundances of bacterial (Figure 2A) and fungal communities (Figure 2B) were determined based on the V1–V3 regions of the 16S rRNA gene and the ITS region, respectively.

Concerning bacterial community structure, staphylococci largely dominated in all sausages. However, a distinct profile for sausages naturally covered with molds was observed as they were mainly colonized by *Staphylococcus equorum* on casing samples, while other *Staphylococcus* spp., including *Staphylococcus saprophyticus*, were most abundant in meat along with *L. sakei* among lactic acid bacteria. Many halotolerant bacterial species were also identified at variable abundances in these samples including those belonging to the *Halomonas*, *Tetragenococcus*, and *Celerinatantimonas* genera. In contrast to these results, *Staphylococcus* sp., *S. saprophyticus*, and *L. sakei* were the three most abundant species in voluntarily inoculated sausages, although *L. sakei* and *S. saprophyticus* were clearly associated with meat samples. Overall, the bacterial communities of these samples were less diversified than those of naturally fermented sausages.

Fungal community structure analyses showed that sausages were largely dominated by the yeast *D. hansenii*, as this species was detected at high abundances in all meat and casing samples. However, other yeast species corresponding to *Kurtzmaniella* (*Kurtzmaniella zeylanoides*), *Candida* (e.g., *Candida parapsilosis* and *Candida sake*), *Yamadazyma* (*Yamadazyma triangularis*), *Wickerhamomyces* (*Wickerhamomyces anomalus*), and *Kazachstania* (*Kazachstania exigua*) were also identified. Among the identified filamentous fungi, *Penicillium* spp. dominated, with *P. nalgiovense* identified at higher abundances in voluntarily inoculated sausages vs. *P. salamii* in the naturally fermented sausages. *Aspergillus* sp., *Trichosporon* sp., and, to a lesser extent, *Alternaria* sp. were also sporadically observed in both meat and casing samples. Chao1 diversity analyses showed that community structure only differed according to sausage weight (*P* = 0.0073) and conservation time (*P* = 0.046) (Supplementary Figure 3). Beta diversity analyses, based on PCoA Bray–Curtis distance analyses at the species level, indicated that fungal communities were grouped together as a function of sample type (i.e., meat or surface, Adonis test, *P* = 0.0377, *R*^2^ = 0.0321), casing type (Adonis test, *P* = 0.0003, *R*^2^ = 0.182), weight (Adonis test, *P* = 0.0003, *R*^2^ = 0.154), or inoculation method (Adonis test, *P* = 0.0003, *R*^2^ = 0.115) (Supplementary Table 3).

### Mycotoxin Determination in Dry-Cured Sausages During Storage

**FIGURE 3:**
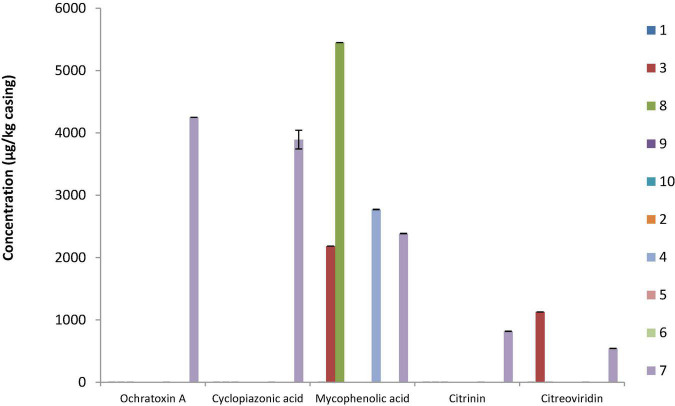
Mycotoxin quantification for the 10 dry fermented sausage casing samples. Results are expressed as micrograms mycotoxin per kilogram casing for average values obtained from three independent fermentation batches. Samples S1, S3, S8, S9, and S10 were sausages voluntarily inoculated with commercial molds, while S2, S4, S5, S6, and S7 were sausages naturally colonized by in-house mycobiota.

Mycotoxin content was determined on the casings of all sausages at different stages during storage (start, middle, and end), and migration into the meat was determined when casing samples were positive. None of the targeted mycotoxins were detected at the start or middle of storage. At the end of storage, one to five mycotoxins could be simultaneously quantified (Figure 3). On the casing of sausage S7, five mycotoxins (OTA, CPA, MPA, CIT, and CTV) were detected at quantifiable levels. This was the only sausage containing OTA, CPA, and CIT. Migration of these mycotoxins into the first 0.5 cm portion of the meat was then evaluated, and while no OTA, MPA, or CTV was detected, CIT and CPA diffused into the meat and reached levels of ∼1,192 ng/g meat and ∼22,236 ng/g meat, respectively. S7 was a sausage naturally colonized with molds, displaying the longest conservation scenario (120 days, 18°C), no protective packaging, and a natural chaudin casing. MPA was the most frequently quantified mycotoxin as it was found on the surface of two naturally surface-colonized (S4 and S7) and two voluntarily surface-inoculated (S3 and S8) sausages; however, no MPA migration was found in any corresponding meat sample (data not shown). For the industrial sausage S3, in which CTV was also detected on the natural chaudin casing, no migration was observed (data not shown). On the contrary, CIT was quantified in S4 and S7 meat samples (1,123 and 1,192 ng/g meat, respectively) and CPA in S3, S7, and S8 meat samples (24,492, 22,236, and 4,432 ng/g meat, respectively). Interestingly, mycotoxin diffusion was observed for three out of four sausages. The latter (S3, S4, and S7) were made with natural chaudin casings and used no or macroperforated packaging, while S8, for which no migration was observed had a collagen casing, was conserved under protective atmosphere and had a long conservation scenario (120 days at 18°C).

### Challenge Tests in Pilot-Scale Fermented Sausage Production Conditions

Among the fungal isolates collected and identified during this study from sausage casings, 14 potentially mycotoxigenic *Penicillium* isolates belonging to five species were preselected based on multiple criteria (species, frequency, morphotype according to the Pitt method, number of sausages containing the species, and known mycotoxin production trait). They included nine *P. nordicum*, two *P. chrysogenum*, one *Penicillium bialowiezense*, one *P. brevicompactum*, and one *P. citreonigrum*. Two *P. nalgiovense* strains (FC35M1 and D5M8) were also included and used for the control condition as they were atoxigenic in the tested conditions. *In vitro* mycotoxin production profiles were first determined after growth on YES medium for the selected strains (*n* = 14) to choose a strong mycotoxin-producing strain for challenge tests. Between one and four mycotoxins were simultaneously produced, and results clearly showed that profiles were strain dependent (Supplementary Figure 4). All *P. nordicum* strains produced OTA. *P. chrysogenum* strains simultaneously produced the highest number of extrolites (AND, MELEA, MPA, and ROQC), while *P. brevicompactum* and *P. bialowiezense* only produced MPA. Based on these profiles, two OTA-producing *P. nordicum* strains (F9M9 and D7M3) were chosen as, unlike other identified mycotoxins, OTA is regulated in some food products.

Challenge tests were then carried out after inoculating equivalent spore suspensions of either the two selected *P. nalgiovense* or *P. nordicum* strains onto the sausage surface. In order to determine whether sausage casing could impact mycotoxin production, pilot-scale sausages were produced using either collagen or natural chaudin casings.

For pilot-scale sausage productions, a loss of 38–40% of sausage weight was observed during drying regardless of the inoculated species. This was expected for this type of sausage, and no major difference was obtained between natural and collagen casings. The pH of fermented sausages typically decreased during ripening (−0.5 to −0.6 pH units) and then increased during drying, with mean values at D30 being between 5.8 and 6.1 pH units. Water activities also progressively lowered during drying (values between 0.872 and 0.901) for all sausages regardless of casing type; however, this difference increased at the end of storage (D50) between the more permeable natural sausage casings and collagen casings with *a*_*w*_ values lowered by 16 vs. 1.4%, respectively. All physicochemical characteristics of pilot-scale dry sausages are provided in Supplementary Table 5.

Lactic acid bacteria are traditionally used as starters in industrially fermented dry sausages, and the commercial bacterial starter cultures used here included *L. sakei* in addition to *S. carnosus*, which were inoculated around 6 log_10_ CFU/g at D0.

**FIGURE 4:**
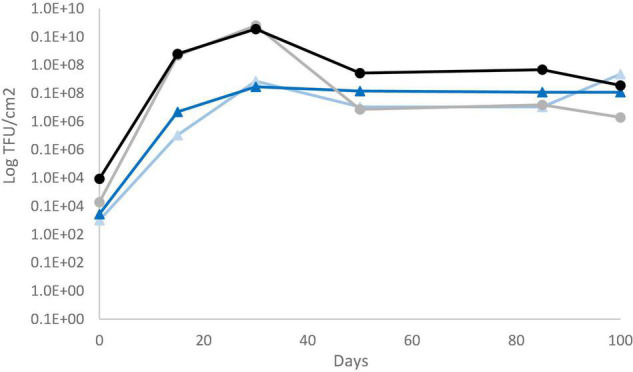
Evolution of *P. nalgiovense* and *P. nordicum* populations during pilot-scale fermented sausage productions using challenge tests. *P. nalgiovense* growth on collagen (gray circles and line) and natural chaudin (black circles and line) casings and *P. nordicum* growth on collagen (light blue circles and line) and natural chaudin (dark blue circles and line) casings.

**FIGURE 5:**
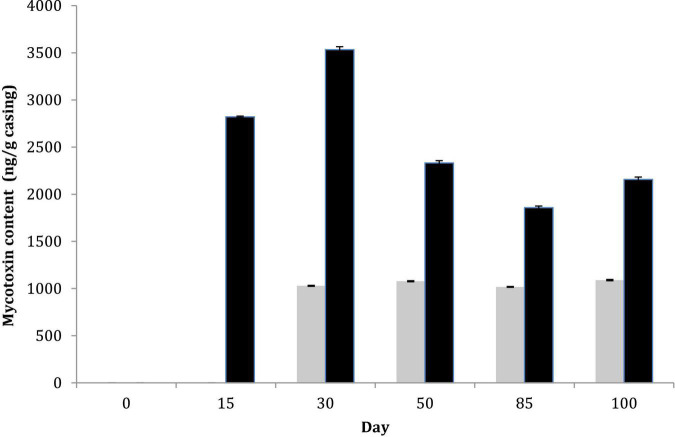
Mycotoxin content during pilot-scale fermented sausage productions using challenge tests after inoculation of two *P. nordicum* isolates on collagen (black) and natural chaudin (gray) casings. No mycotoxins were produced by *P. nalgiovense* and therefore omitted from the figure.

Total aerobic mesophilic and LAB counts were monitored, and similar values were obtained for both sausage productions, regardless of casing type. LAB counts had a significant increase from about 6 log_10_ CFU g^–1^ (concentration of the inoculum) to 8 log_10_ CFU g^–1^ over the first days of ripening. Then, LAB populations remained relatively constant over the drying process and the subsequent storage, regardless of the casing type (natural vs. collagen casing) (Supplementary Figure 5). Fungal counts were also monitored at six sampling dates. For *P. nalgiovense*-inoculated products, fungal counts were systematically higher on natural chaudin casings than on collagen casings during conservation. However, both *P. nalgiovense* and *P. nordicum* grew abundantly on both casings, and their populations remained abundant until the end of conservation (Figure 4). In parallel to these analyses, mycotoxin content was also determined at the same sampling times. No mycotoxins were detected in sausages produced with the *P. nalgiovense* strains, while OTA was detected for sausages produced with *P. nordicum* strains (Figure 5). The highest levels were observed between 15 and 30 days (∼2,700 to 3,500 ng/g casing) for sausages produced with collagen casings and then decreased and remained relatively stable (∼2,000 ng/g casing) between 50 and 100 days. For natural chaudin casings, OTA was detected at 30 days at ∼1,000 ng/g casing and remained stable during the entire conservation time.

## Discussion

In this study, microbial diversity, with a special focus on fungal species including potentially mycotoxigenic ones, was determined on fermented sausages that were naturally or voluntarily surface inoculated with molds.

For bacterial communities, staphylococci systematically dominated with higher abundances of *S. equorum* in naturally surface-inoculated fermented sausages vs. *S. saprophyticus* in voluntarily fermented sausages followed by lactic acid bacteria species, especially in meat. These are commonly encountered as the most prevalent species in South European dry fermented sausages and strongly contribute to their sensory properties (Van Reckem et al., 2019, 2020). The contribution of lactic acid bacteria relies on the production of organic acids and volatile compounds, whereas coagulase-negative staphylococci are responsible for color development and stabilization, proteolysis, lipolysis, and decomposition of free amino acids and peroxides (Aquilanti et al., 2016). The manufacture of dry fermented sausages is usually initiated by using a commercial starter culture, which frequently includes *L. sakei* in combination with coagulase-negative staphylococci species (e.g., *S. xylosus* and/or *S. carnosus*) (Ojha et al., 2015), but in the present study, details about the processing conditions were not known. Halotolerant bacterial species were also identified in the majority of samples and included *Halomonas* spp., *Tetragenococcus* spp., and *Celerinatantimonas* spp. already described in diverse salty to highly salty fermented foods (i.e., cheese, table olives, soy sauce, fish sauce, soybean paste, etc.). These bacterial genera participate to the fermentation process and can contribute to the sensory characteristics of the fermented foods (Sitdhipol et al., 2013; Amadoro et al., 2015; Yunita and Dodd, 2018; Penland et al., 2020; Sawada et al., 2021).

For fungi, only slight quantitative variations in fungal populations were observed between samples during conservation (up to a 2.6 log_10_ TFU/cm^2^). However, a difference in yeast-to-mold ratios was observed, especially at the start of the conservation period with higher yeast counts observed for naturally fermented sausages. These differences were not directly linked to any of the measured physicochemical parameters of the sausages. The observed differences appeared to be specific to each producer and are thus associated with the specific technological parameters applied during production (temperature, relative humidity, drying time, etc.). Fungal diversity of voluntarily inoculated fermented sausages showed lower species diversity, as expected. The most abundant species identified by both culture-dependent and -independent analyses, namely, *P. nalgiovense*, *P. nordicum*, and *D. hansenii*, are among the most commonly encountered mycobiota on sausage casings, although other *Penicillium* spp. including *P. chrysogenum*, *P. olsonii*, *P. solitum*, or *P. salamii* are also frequent (Leistner, 1990; Andersen, 1995; López-Díaz et al., 2001; Sunesen and Stahnke, 2003; Ludemann et al., 2004; Tabuc et al., 2004; Asefa et al., 2010; Perrone et al., 2015; Meftah et al., 2018; Vila et al., 2019). Based on ANOVA, the technological species, *P. nalgiovense*, was clearly associated with voluntarily inoculated sausage casings and most abundant on collagen casings, while *P. salamii* was highly associated with the naturally surface-colonized sausages (*P* = 0.016). *P. nalgiovense* is the most well-known technological species used to produce dry cured meats. It has been proven to be a highly effective starter to colonize sausage casings (creating the desired white or whitish-gray velvety mycelial covering), prevent growth of unwanted contaminating fungal species, and enhance the overall organoleptic quality of the final product (Leistner, 1990; Martin et al., 2006; Iacumin et al., 2009; Ludemann et al., 2009), thus explaining its commercial production for direct inoculation. Noteworthy, its ability to potentially produce penicillin should also be considered (Laich et al., 2003), and strains selected for industrial use should preferentially lack this trait. The atoxigenic *P. salamii* species was also recently described as a very promising starter culture due to its efficient ability to colonize salami-style sausages (Perrone et al., 2015; Magista‘ et al., 2017). Interestingly, this species co-dominated the naturally surface-colonized sausages, thus suggesting that it is part of the natural mycobiota of French dry cured meats. Further studies would be of interest to determine whether this species could be used as a starter by determining whether it positively impacts the overall sensorial properties of French fermented sausages. During this study, three *P. salamii* as well as 10 *P. nalgiovense* isolates were actually screened for mycotoxin production, and none of the target extrolites were detected (data not shown), thus confirming their described safety status.

In comparison to the voluntarily inoculated sausages, naturally surface-colonized sausages’ mycobiota were much more diversified and unique, especially at the start of the conservation time (after drying), which is certainly linked to the processing environment and the natural microbiota encountered in the drying chambers. This was shown by both culture-dependent and -independent approaches. The most dominant species belonged to *P. nalgiovense*; *P. nordicum*; and more sporadically to *Scopulariopsis* sp., *D. hansenii*, *C. zeylanoïdes*, and other *Penicillium* species (*P. chrysogenum*, *P. solitum*, *P. brevicompactum*, *P. bialowiezense*, *P. chermisinum*, *P. salamii*, etc.). The systematic presence of *P. nalgiovense* and, among yeasts, *D. hansenii*, reinforces their established technological role during dry-cured meat production. Other species were more sporadically observed and likely correspond to either the in-house mycobiota and/or were contaminating airborne species (i.e., *Scopulariopsis* spp., *C. zeylanoides*, *Cladosporium* spp., *A. fumigatus*, etc.). The presence of these minor contaminating fungal species suggests their ability to be transferred from the working environment to the products. They are also more xerotolerant than other fungal species such as *P. nalgiovense*, which could explain why they colonize the surface when *a*_*w*_ decreases, and the *P. nalgiovense* barrier effect may not be as efficient during this stage due to the decrease in sausage humidity. *Scopulariopsis* species are ubiquitous and frequently encountered in diverse fermented foods (e.g., cheese and meats) (Ropars et al., 2012) as well as human and environmental niches (e.g., indoors and wastewaters) (Jagielski et al., 2016; Woudenberg et al., 2017), so it can also be expected to be part of the mycobiota of fermented meats.

The in-house mycobiota of naturally fermented meats have already been documented for traditional products around the world (López-Díaz et al., 2001; Ludemann et al., 2004; Iacumin et al., 2009; Vila et al., 2019) and can be linked to the presence of diversified environmental fungal species as observed in this study. *Cladosporium* spp. are described as common contaminants in dry-cured meats and known to provoke unpleasant black spots on the surface (Lozano-Ojalvo et al., 2015; Alía et al., 2016). Other fungal species such as some *Penicillium* spp. may also cause coloration problems like unwanted green or gray surface spots or covering (Pitt and Hocking, 2009; Asefa et al., 2010); however, non-uniformly white sausage surfaces are not necessarily considered as a defect for more traditionally and naturally produced fermented meats as part of their product typicity.

For *P. nalgiovense*, although 16 different morphotypes were observed, only two morphotypes were identified from sausages produced by at least two different producers. This clearly highlighted the phenotypic diversity within this species, which could be of interest during strain selection for distinct traits. Conidia varied in color on CYA plates from brownish to green to white, although all the known industrial molds included in this study exhibited a white phenotype. *Aspergillus* are also frequent (airborne) contaminants in fermented meats and include many potentially mycotoxigenic species. They are frequently identified among in-house fungal genera spontaneously colonizing sausage casings of naturally fermented meats (Perrone et al., 2015). In this study, they were only sporadically identified at low levels in some batches, and no *Aspergillus*-related mycotoxins (aflatoxins) were detected. Besides mycotoxins, *Aspergillus ochraceus* was previously shown to produce an undesirable yellowish gold discoloration on sausage casings, which can clearly lead to economic losses for producers due to product spoilage (Canel et al., 2013); however, no such observations were made for the studied sausages.

Mycotoxin risk was then evaluated to link the presence of potentially mycotoxigenic molds identified on sausage casings to actual mycotoxin production in samples. Extrolites were therefore extracted from sausage casings and, if detected, diffusion into meat was evaluated just below the surface. This study revealed a relatively low level of mycotoxins, especially those known to have the highest toxicities, even though 40% of samples contained one or more mycotoxins at the end of conservation while none were detected at the start or middle of the conservation time. In positive samples, multi-mycotoxin contaminations were observed (up to five metabolites), with a higher prevalence in sausages made with natural casings. These mycotoxins are well known to be produced by different *Penicillium* species, *P. nordicum* being able to produce OTA and *P. chrysogenum*, MPA. Migration of two mycotoxins, namely, CIT and CPA, was also observed into the meat, thus implying that mycotoxins can diffuse below the casing surface. Also, the presence of *D. hansenii* on casings could have an impact as it is considered as a competitive bioprotective species against toxigenic penicillia in dry-cured meats, potentially reducing mycotoxin levels in samples (Núñez et al., 2015; Murgia et al., 2019; Peromingo et al., 2019a). Both mycotoxin production and migration into foods will depend on different factors, namely, biological (mold species/strain, mycotoxin properties, etc.), storage (temperature and time), and product (*a*_*w*_, pH, composition, etc.)-related factors (Coton and Dantigny, 2019). Regarding mycotoxin migration, to date, only a few studies have shown that this can occur in sausages contaminated by either OTA-producing *Aspergillus ochraceus* (Escher et al., 1973) or *Aspergillus westerdijkiae* (Parussolo et al., 2019) or mycotoxin-producing *Penicillium* spp. (Escher et al., 1973; Peromingo et al., 2019b). However, further studies are required to better understand this phenomenon, and both depthwise and widthwise diffusion into dry-cured meats should be evaluated using a worst-case-scenario approach (Coton et al., 2019, 2020) in order to be able to provide sound guidelines to consumers. In comparison, Iacumin et al. (2009) studied 160 traditionally and industrially fermented Italian dry-cured sausages and showed that 45% of casing samples contained quantifiable amounts of OTA ranging from 3 to 18 μg/kg (37/100 traditional and 35/60 industrial sausage casings); however, no OTA was detected in the meat. In Italian Salame Piemonte, multiple batches produced in the same factory in different months were analyzed for mycotoxin content, and only low levels of roquefortine C were identified on casings despite the presence of potentially mycotoxigenic *Penicillium* spp. (Franciosa et al., 2021). In France, only one study has been done to characterize fungal diversity on dry-cured sausages and determine *in vitro* mycotoxin potential (Tabuc et al., 2004). Only five sausages were studied and 13 fungal strains isolated. The obtained results showed that three *P. viridicatum* strains produced CPA at concentrations up to 12 mg/kg, but no details on mycotoxin content in the meat was given.

In pilot-scale dry sausage challenge tests, no mycotoxins were detected in sausages produced by *P. nalgiovense*, again confirming the safety status of this technological starter. On the other hand, collagen casing sausages produced with the two selected OTA-producing *P. nordicum* strains had the highest OTA contents compared to the more permeable chaudin casing. However, it should be emphasized that this challenge test was carried out using worst-case-scenario conditions with the highest mycotoxin-producing strains identified in our study and after inoculating the sausage casings with a highly concentrated spore suspension. Moreover, no biotic competition intervened as no commercial culture was added. In this study, OTA levels progressively and concomitantly increased with *P. nordicum* counts on collagen casings (during fungal colonization on the casing surface), then decreased and stabilized for the remainder of the conservation time, which could be linked to the sudden decrease in *a*_*w*_ values. OTA may have also potentially diffused into the meat, but no analyses were done on meat samples in this part of the study to confirm this hypothesis. OTA production by *P. nordicum* was previously shown in artificially inoculated dry-cured pork-based medium or dry-cured pork cores, and production was strongly influenced by both temperature (higher at 20°C) and *a*_*w*_ parameters (higher at 0.93) (Battilani et al., 2007). Sánchez-Montero et al. (2019) also showed that *P. nordicum* and *Penicillium verrucosum* can produce high concentrations of OTA after inoculation onto dry-cured ham and dry-cured sausage slices. Both *P. nordicum* and *P. verrucosum* have been described to be well adapted to salt-rich environments, including dry-cured meats (Schmidt-Heydt et al., 2012; Geisen et al., 2017, 2018). Interestingly, *P. nordicum* was shown to be a strong OTA producer over a wide range of NaCl concentrations (from 2 to 100 g/l with a weak optimum at 20 g/l in YES agar), while *P. verrucosum* was shown to shift from CIT production at lower NaCl concentrations to OTA at higher ones (Schmidt-Heydt et al., 2012). It appears that both biosynthesis and excretion of OTA play a role in fungal cell chloride homeostasis (Schmidt-Heydt et al., 2012) as OTA contains a chloride atom in its chemical structure, thus suggesting why these species are so well adapted and prevalent in dry-cured meats among other salt-rich foods.

Based on the results of this study, a highly diversified mycobiota was clearly identified for dry fermented sausages naturally surface colonized with in-house mycobiota in comparison to voluntarily surface-inoculated dry fermented sausages. This included the presence of mycotoxigenic species, although not systematically identified and usually subdominant in comparison to the typical technological fungal species *P. nalgiovense*, *D. hansenii*, or even *P. salamii*. Mycotoxin content was evaluated, and the actual risk associated with potential acute toxicity for consumers is relatively low as the incidence of mycotoxins identified among samples was low and mostly concentrated on the sausage casings (often removed before consumption). Furthermore, based on the tolerable dose for OTA that was set to 120 ng/kg body weight, this value should be considered in relation to what was quantified on casing samples and what a typical consumer might consume when it comes to portions of edible casings. To minimize the potential mycotoxin risk, future studies could be focused on selecting new atoxigenic and non-penicillin-producing fungi that efficiently colonize the sausage casings, exhibit potential bioprotective abilities against other contaminating fungi, and positively impact the sensorial properties of the final product.

## Data Availability Statement

The datasets presented in this study can be found in online repositories. The names of the repository/repositories and accession number(s) can be found below: https://www.ncbi.nlm.nih.gov/, Bioproject ID PRJNA757247.

## Author Contributions

MC, BF, FD, and EC obtained the funding and supervised the study. MC, BF, and FD designed the experiments. RJ and AP performed all experimental work related to fungi and extrolite extractions, while ER performed microbiological and physicochemical analyses of sausages. RJ and AP extracted all DNA samples for metabarcoding analyses, while JM, BT, MC, and GD were involved in metabarcoding and statistical analyses. BF, ER, and SJ performed pilot-scale sausage productions and bacterial and physicochemical analyses of sausages. MC, RJ, and AP did all LC-MS data acquisitions, and MC performed mycotoxin quantification analyses. MC drafted the manuscript. All authors contributed to the article and approved the submitted version.

## Conflict of Interest

The authors declare that the research was conducted in the absence of any commercial or financial relationships that could be construed as a potential conflict of interest.

## Publisher’s Note

All claims expressed in this article are solely those of the authors and do not necessarily represent those of their affiliated organizations, or those of the publisher, the editors and the reviewers. Any product that may be evaluated in this article, or claim that may be made by its manufacturer, is not guaranteed or endorsed by the publisher.

## Footnotes

1
https://www.fict.fr/notre-industrie/chiffres-cles/

2
https://github.com/torognes/vsearch

3
https://rdp.cme.msu.edu/seqmatch

4
http://www.ncbi.nlm.nih.gov/BLAST

5
https://submit.ncbi.nlm.nih.gov/subs/sra/
