# Supplementary material for: Microbial Ecology of French Dry Fermented Sausages and Mycotoxin Risk Evaluation During Storage
Source: Front Microbiol. 2021 Nov 4;12:737140. doi: 10.3389/fmicb.2021.737140 (PMC8601720; doi:10.3389/fmicb.2021.737140)
Supplement: Supplementary file 1 [file Table_1.docx]

**Table S1.** Method performance characteristics for metabolite quantification from fermented sausages.

| **Compound** | **Formula** | **RT (min)** | **Quantifier Ion (Q1) (m/z)** | **Qualifier Ion (Q2) (m/z)** | **R^2^** | **DL**  **(ng.g^-1^)** | **QL**  **(ng.g^-1^)** | **ESI** |
| --- | --- | --- | --- | --- | --- | --- | --- | --- |
| PAT | C_7_H_6_O_4_ | 0.8 | 153.0193 | 177.0193 | 0.999 | 6405.01 | 19409.12 | - |
| ISOFUMI A | C_18_H_22_N_2_O_2_ | 2.3 | 299.1751 | 322.1573 | 0.996 | 23.31 | 76.70 | + |
| CIT | C_13_H_14_O_5_ | 10.9 | 251.0914 | 273.0733 | 0.998 | 72.35 | 219.26 | + |
| EREM B | C_15_H_20_O_3_ | 11.4 | 249.1484 | 271.1252 | 0.999 | 82.91 | 251.24 | + |
| MELEA | C_23_H_23_N_5_O_4_ | 12.5 | 434.1823 | 456.1642 | 0.998 | 56.47 | 171.13 | + |
| AFLB1 | C_17_H_12_O_6_ | 13.2 | 313.0707 | 335.0526 | 0.994 | 37.39 | 113.29 | + |
| ROQ C | C_22_H_23_N_5_O_2_ | 15.1 | 390.1928 | N/A | 0.977 | 136.78 | 414.47 | + |
| EREM A | C_17_H_22_O_5_ | 16.1 | 307.1561 | 329.1358 | 0.999 | 124.46 | 377.16 | + |
| GRISEO | C_17_H_17_ClO_6_ | 17.9 | 353.0786 | 375.0606 | 0.991 | 30.35 | 91.96 | + |
| MPA | C_17_H_20_O_6_ | 18.1 | 321.1334 | 303.1231 | 0.999 | 121.47 | 368.10 | + |
| CITREO | C_23_H_30_O_6_ | 19.6 | 403.2115 | 425.1935 | 0.999 | 81.83 | 247.97 | + |
| OTA | C_20_H_18_ClNO_6_ | 21.1 | 404.0895 | 426.0715 | 0.995 | 148.31 | 449.44 | + |
| STERIG | C_18_H_12_O_6_ | 21.9 | 325.0707 | 347.0526 | 0.999 | 61.13 | 185.24 | + |
| CPA | C_20_H_20_N_2_O_7_ | 23.3 | 337.1547 | 359.1366 | 0.997 | 112.80 | 341.82 | + |
| AND A | C_28_H_38_O_7_ | 23.5 | 485.2541 | N/A | 0.999 | 102.86 | 311.70 | - |
| PEN A | C_37_H_44_ClNO_6_ | 29.1 | 632.2781 | N/A | 0.993 | 198.89 | 602.71 | - |
| PR toxin | C_17_H_20_O_6_ | NA | 321.1332 | 343.1148 | 0.996 | NA | NA | + |

RT: Retention Time; R^2^: correlation coefficient; DL: Detection Limit; QL: Quantification Limit; ESI: Electrospray Ionization; NA: not applicable

| Fermented dry sausage | Sample  type | Sampling date | pH | *a_w_* | Lactic acid bacteria  log CFU/g | TAM  log CFU/g |
| --- | --- | --- | --- | --- | --- | --- |
| S1 | Casing (C*) | D_0_ | 5.89 ± 0.45 | 0.865 ± 0.027 | 6.63 ± 0.70 | 7.71 ± 0.30 |
|  |  | D_End_ | 6.93 ± 0.25 | 0.829 ± 0.037 | 2.29 ± 0.18 | 8.42 ± 0.49 |
|  | Meat | D_0_ | 4.87 ± 0.12 | 0.880 ± 0.011 | 8.09 ± 0.50 | 8.06 ± 0.57 |
|  |  | D_End_ | 5.89 ± 0.37 | 0.834 ± 0.036 | 6.27 ± 0.59 | 6.59 ± 0.27 |
| S3 | Casing (N) | D_0_ | 5.65 ± 0.12 | 0.846 ± 0.017 | 6.31 ± 0.33 | 8.30 ± 0.29 |
|  |  | D_End_ | 6.38 ± 0.30 | 0.781 ± 0.018 | 4.37 ± 0.80 | 8.39 ± 0.49 |
|  | Meat | D_0_ | 5.14 ± 0.11 | 0.882 ± 0.007 | 8.46 ± 0.40 | 8.65 ± 0.14 |
|  |  | D_End_ | 5.73 ± 0.13 | 0.805 ± 0.022 | 6.41 ± 0.67 | 6.77 ± 0.46 |
| S8 | Casing (C) | D_0_ | 5.63 ± 0.16 | 0.759 ± 0.042 | 7.37 ± 0.31 | 7.51 ± 0.22 |
|  |  | D_End_ | 5.68 ± 0.05 | 0.689 ± 0.012 | 5.20 ± 0.29 | 4.93 ± 0.42 |
|  | Meat | D_0_ | 5.58 ± 0.17 | 0.761 ± 0.041 | 8.27 ± 0.18 | 8.19 ± 0.11 |
|  |  | D_End_ | 5.68 ± 0.05 | 0.715 ± 0.005 | 6.00 ± 0.46 | 6.45 ± 0.41 |
| S9 | Casing (C) | D_0_ | 6.50 ± 0.35 | 0.850 ± 0.014 | 7.58 ± 0.17 | 7.10 ± 0.15 |
|  |  | D_End_ | 5.96 ± 0.34 | 0.780 ± 0.020 | 0.61 ± 0.78 | 2.87 ± 0.45 |
|  | Meat | D_0_ | 5.68 ± 0.13 | 0.851 ± 0.010 | 7.73 ± 0.16 | 7.91 ± 0.15 |
|  |  | D_End_ | 5.79 ± 0.21 | 0.827 ± 0.004 | 4.54 ± 0.34 | 5.62 ± 0.28 |
| S10 | Casing (C) | D_0_ | 5.71 ± 0.59 | 0.874 ± 0.030 | 6.11 ± 0.32 | 6.92 ± 0.23 |
|  |  | D_End_ | 7.13 ± 0.18 | 0.839 ± 0.047 | 3.58 ± 1.15 | 8.34 ± 0.24 |
|  | Meat | D_0_ | 5.11 ± 0.27 | 0.880 ± 0.027 | 7.80 ± 0.11 | 7.90 ± 0.46 |
|  |  | D_End_ | 6.64 ± 0.36 | 0.858 ± 0.036 | 5.28 ± 0.73 | 6.63 ± 0.29 |
| S2 | Casing (N) | D_0_ | 5.50 ± 0.39 | 0.866 ± 0.041 | 6.81 ± 0.38 | 8.72 ± 0.64 |
|  |  | D_End_ | 6.45 ± 0.28 | 0.708 ± 0.043 | 5.34 ± 0.95 | 8.90 ± 0.18 |
|  | Meat | D_0_ | 5.19 ± 0.10 | 0.902 ± 0.027 | 9.03 ± 0.11 | 9.16 ± 0.08 |
|  |  | D_End_ | 6.19 ± 0.23 | 0.756 ± 0.034 | 6.75 ± 0.30 | 6.99 ± 0.58 |
| S4 | Casing (N) | D_0_ | 6.23 ± 0.37 | 0.826 ± 0.012 | 6.34 ± 0.46 | 9.05 ± 0.19 |
|  |  | D_End_ | 7.59 ± 0.16 | 0.749 ± 0.038 | 1.45 ± 0.20 | 9.34 ± 0.32 |
|  | Meat | D_0_ | 5.77 ± 0.16 | 0.838 ± 0.008 | 8.18 ± 0.13 | 8.38 ± 0.13 |
|  |  | D_End_ | 7.01 ± 0.14 | 0.800 ± 0.027 | 5.66 ± 0.28 | 6.35 ± 0.27 |
| S5 | Casing (N) | D_0_ | 5.91 ± 0.19 | 0.738 ± 0.018 | 6.22 ± 0.06 | 8.41 ± 0.18 |
|  |  | D_End_ | 6.01 ± 0.07 | 0.665 ± 0.011 | 5.26 ± 0.34 | 7.61 ± 0.27 |
|  | Meat | D_0_ | 5.55 ± 0.08 | 0.827 ± 0.022 | 8.67 ± 0.09 | 8.68 ± 0.07 |
|  |  | D_End_ | 5.79 ± 0.05 | 0.705 ± 0.011 | 7.55 ± 0.11 | 7.14 ± 0.18 |
| S6 | Casing (N) | D_0_ | 6.03 ± 0.14 | 0.746 ± 0.018 | 5.63 ± 0.63 | 8.24 ± 0.43 |
|  |  | D_End_ | 5.86 ± 0.14 | 0.664 ± 0.012 | 5.27 ± 0.59 | 7.06 ± 0.54 |
|  | Meat | D_0_ | 5.62 ± 0.15 | 0.823 ± 0.024 | 8.36 ± 0.06 | 8.37 ± 0.11 |
|  |  | D_End_ | 5.71 ± 0.14 | 0.712 ± 0.011 | 7.43 ± 0.20 | 7.25 ± 0.30 |
| S7 | Casing (N) | D_0_ | 5.95 ± 0.06 | 0.820 ± 0.015 | 6.35 ± 1.41 | 7.13 ± 0.89 |
|  |  | D_End_ | 6.10 ± 0.04 | 0.575 ± 0.029 | 5.26 ± 0.56 | 5.80 ± 0.31 |
|  | Meat | D_0_ | 5.70 ± 0.05 | 0.771 ± 0.016 | 8.31 ± 0.19 | 8.35 ± 0.16 |
|  |  | D_End_ | 5.99 ± 0.14 | 0.688 ± 0.003 | 6.54 ± 0.25 | 6.73 ± 0.19 |

**Table S2:** Physicochemical (pH and *a_w_*) and microbiological (lactic acid bacteria and total aerobic mesophilic (TAM) counts) characteristics of the 5 fermented dried sausages voluntarily sprayed with a commercial mold suspension (S1, S3, S8, S9 and S10) or naturally covered with indigenous mycobiota of the processing plants (S2, S4, S5, S6 and S7) (mean value of *n* = 5 samples ± Standard Deviation) at the beginning (D_0_) and end of storage (D_End_). * Casing types are shown in parentheses i.e. C (collagen) or N (natural, “chaudin” or “menu”)

**Table S3**. Adonis Bray-Curtis analyses performed on sample type, fermentation stage, casing type, inoculation mode, packaging conditions, weight and conservation scenario data.


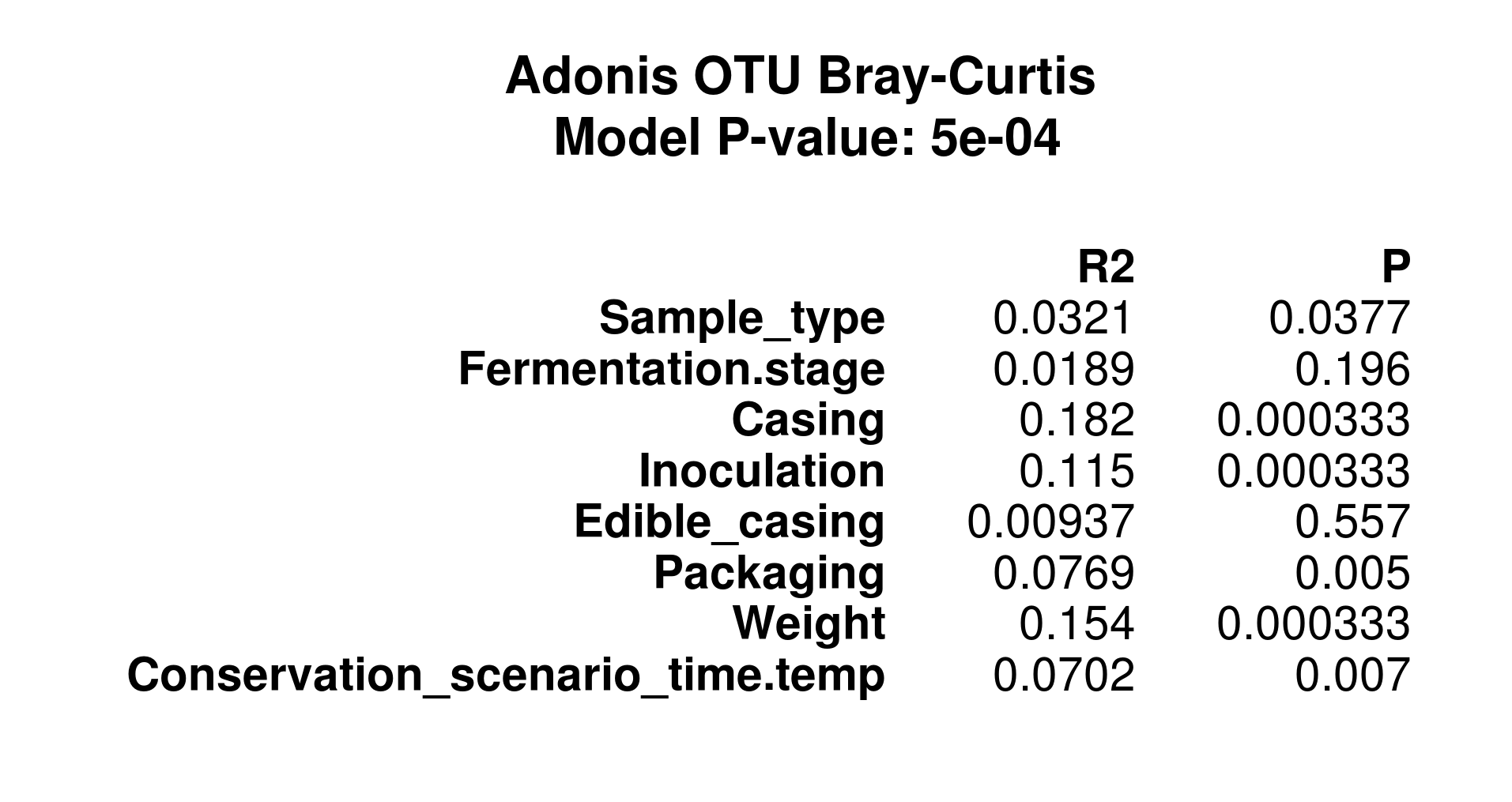


**Table S4:** Ripening and drying parameters used in the present study.

| Time (hour) | Temperature (°C) | Relative humidity (%) |
| --- | --- | --- |
| 3 | 8 | 45 - 96 |
| 1 | 17 | 92 - 96 |
| 6 | 21 | 86 – 93 |
| 20 | 22 – 24 | 82 – 90 |
| 4 | 22 – 24 | 75 – 85 |
| 6 | 20 | 75 – 82 |
| 6 | 17 | 83 – 90 |
| 3 | 16 | 82 – 90 |
| 3 | 14 | 73 – 82 |
| 671 | 13 | 73 – 81 |
| 480 | 18 | 55 – 60 |

**Table S5.** Physicochemical characteristics of *P. nalgiovense*-sprayed or *P. nordicum-*sprayed fermented dry sausages made with natural or collagen casing during ripening/drying processes (from D0 to D30) and subsequent storage (from D30 to D50). Results were based on triplicate analysis.

| Sampling days | Fermented dry sausages with natural casing | | |  | Fermented dry sausages with collagen casing | | |
| --- | --- | --- | --- | --- | --- | --- | --- |
|  | Weight loss (%) | pH | a_w_ |  | Weight loss (%) | pH | a_w_ |
| *P. nalgiovense*-sprayed dry sausages | | | | | | | |
| D0 | NA | 5.89 ± 0.017 | 0.965 ± 0.002 |  | NA | 5.89 ± 0.017 | 0.965 ± 0.002 |
| D3 | -4.21 | 5.43 ± 0.086 | 0.956 ± 0.002 |  | -2.96 | 5.32 ± 0.035 | 0.964 ± 0.001 |
| D15 | -26.23 | 6.10 ± 0.061 | 0.932 ± 0.007 |  | -24.85 | 5.77 ± 0.159 | 0.948 ± 0.002 |
| D30 | -39.47 | 6.05 ± 0.125 | 0.872 ± 0.016 |  | -38.17 | 5.87 ± 0.214 | 0.899 ± 0.004 |
| D50 | ND | 6.06 ± 0.095 | 0.748 ± 0.022 |  | ND | 5.84 ± 0.086 | 0.878 ± 0.005 |
| *P. nordicum*-sprayed dry sausages | | | | | | | |
| D0 | NA | 5.83 ± 0.031 | 0.966 ± 0.004 |  | NA | 5.83 ± 0.031 | 0.966 ± 0.004 |
| D3 | -7.46 | 5.30 ± 0.039 | 0.962 ± 0.007 |  | -4.80 | 5.27 ± 0.023 | 0.971 ± 0.003 |
| D15 | -28.27 | 5.86 ± 0.059 | 0.930 ± 0.013 |  | -24.46 | 5.52 ± 0.299 | 0.942 ± 0.006 |
| D30 | -39.48 | 5.99 ± 0.179 | 0.884 ± 0.011 |  | -38.17 | 5.84 ± 0.243 | 0.901 ± 0.013 |
| D50 | ND | 5.87 ± 0.162 | 0.731 ± 0.029 |  | ND | 5.88 ± 0.209 | 0.897 ± 0.018 |
