# Supplementary material for: Microbial Ecology of French Dry Fermented Sausages and Mycotoxin Risk Evaluation During Storage
Source: Front Microbiol. 2021 Nov 4;12:737140. doi: 10.3389/fmicb.2021.737140 (PMC8601720; doi:10.3389/fmicb.2021.737140)

**Supplementary Figure S1**. Yeast and mold counts on sausage casings at the start and end of conservation, for the dry fermented sausages voluntarily (1, 3, 8, 9, 10) or naturally (2, 4, 5, 6, 7) surface-inoculated with molds. Dark and light blues correspond to molds at the start and end of conservation, respectively. Dark and light greens correspond to yeast at the start and end of conservation, respectively_._


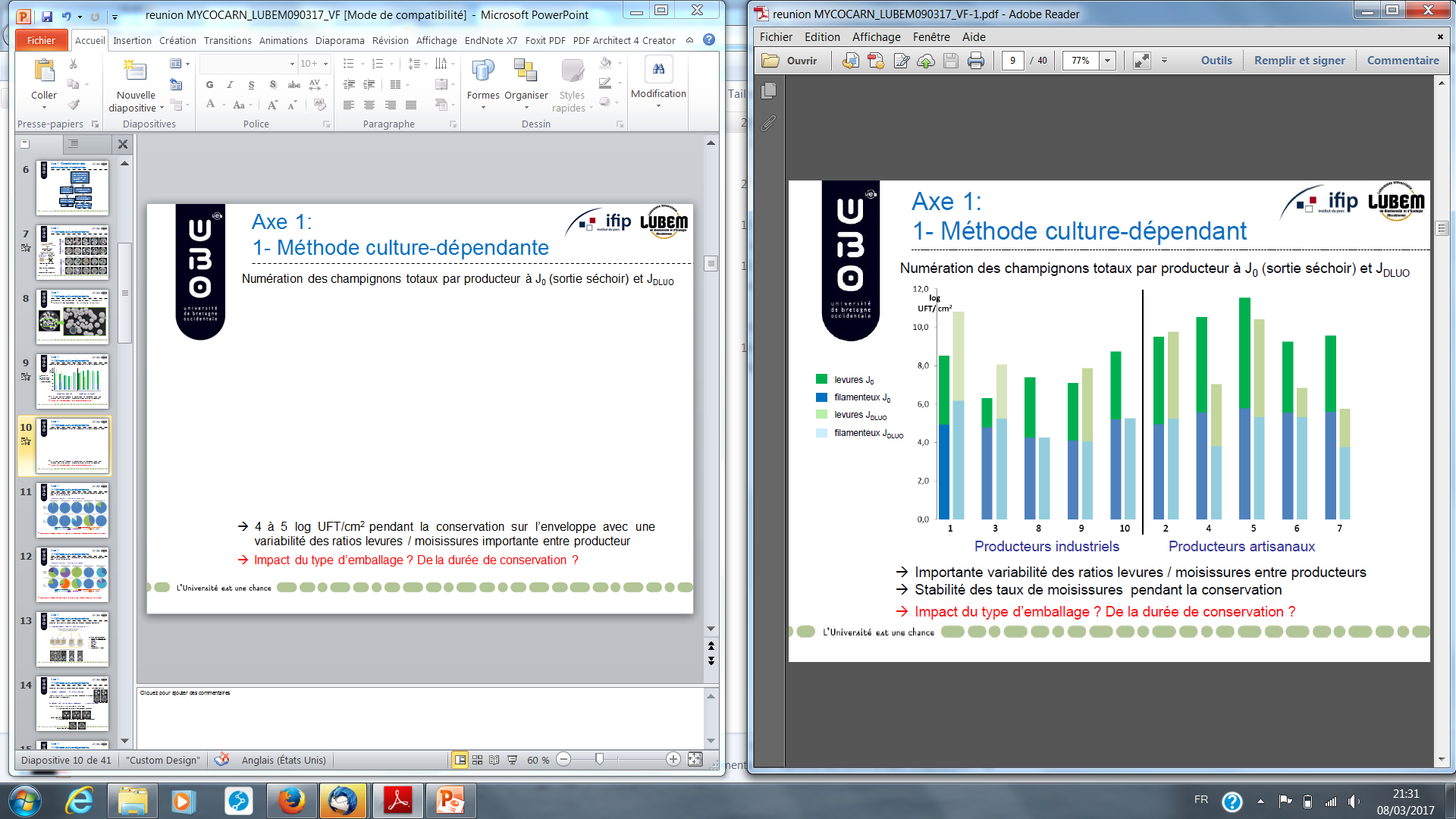



**Supplementary Figure 2**. Examples of different morphotypes observed after 7-days growth on CYA at 25°C according to the Pitt method for *P. nalgiovense* (A), *P. nordicum* (B) and *P. chrysogenum* (C) isolates

A


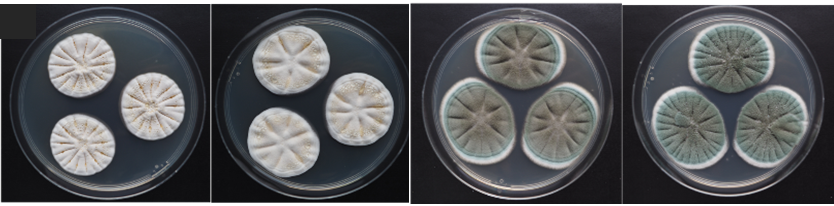


B


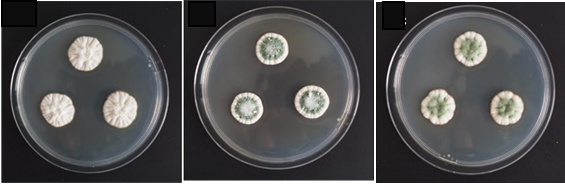


C


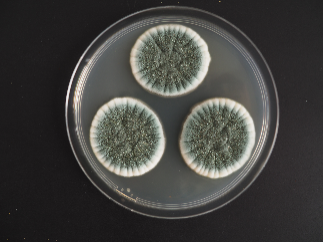

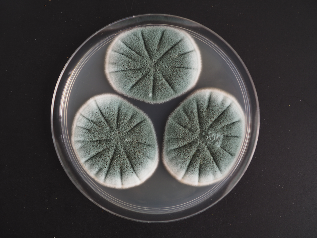


**Supplementary Figure 3**. Chaos1 diversity analyses on samples rarefied to a read depth of 1054. Stripchart presentation for weight (A) and conservation time (B)

A


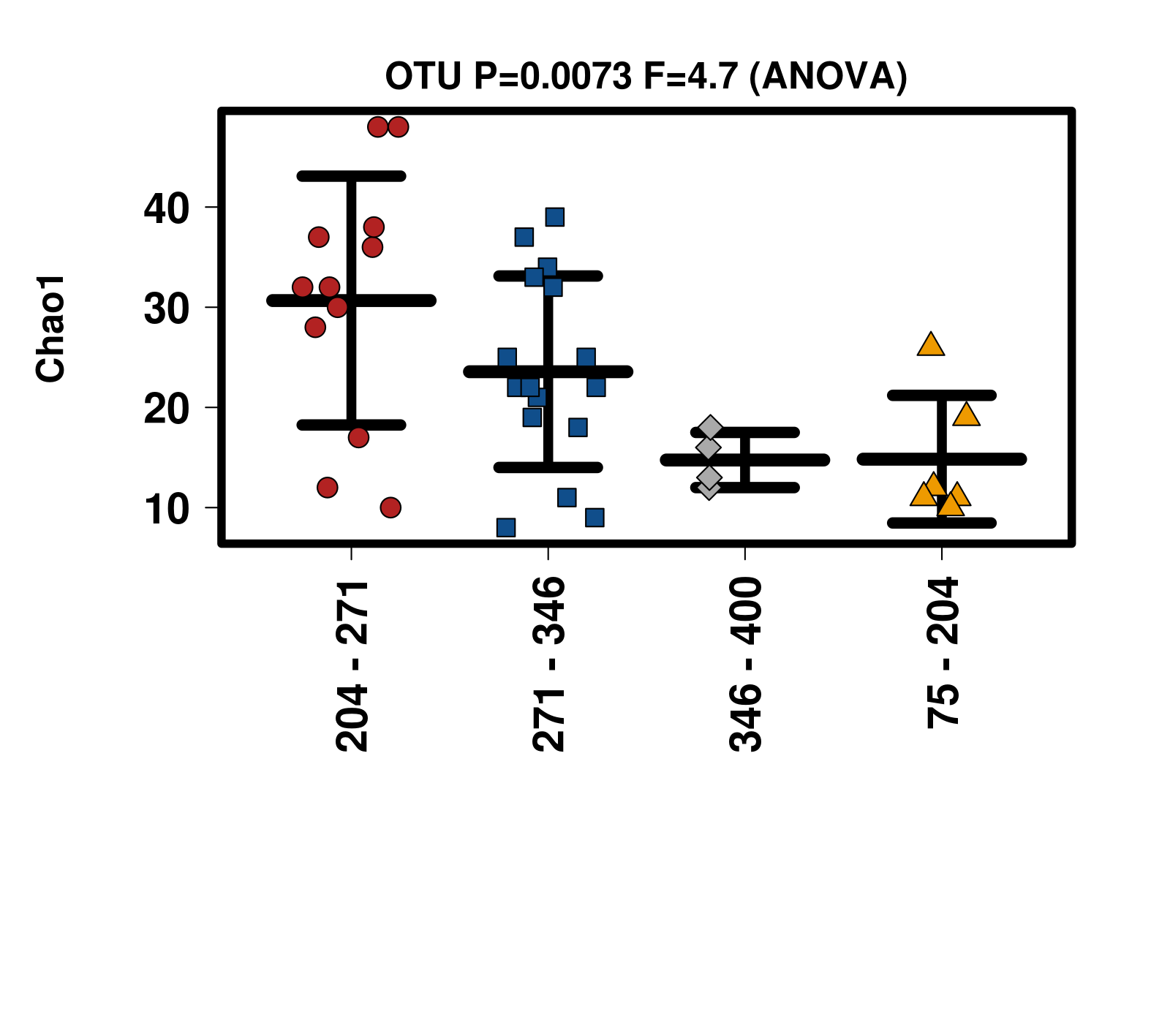


B


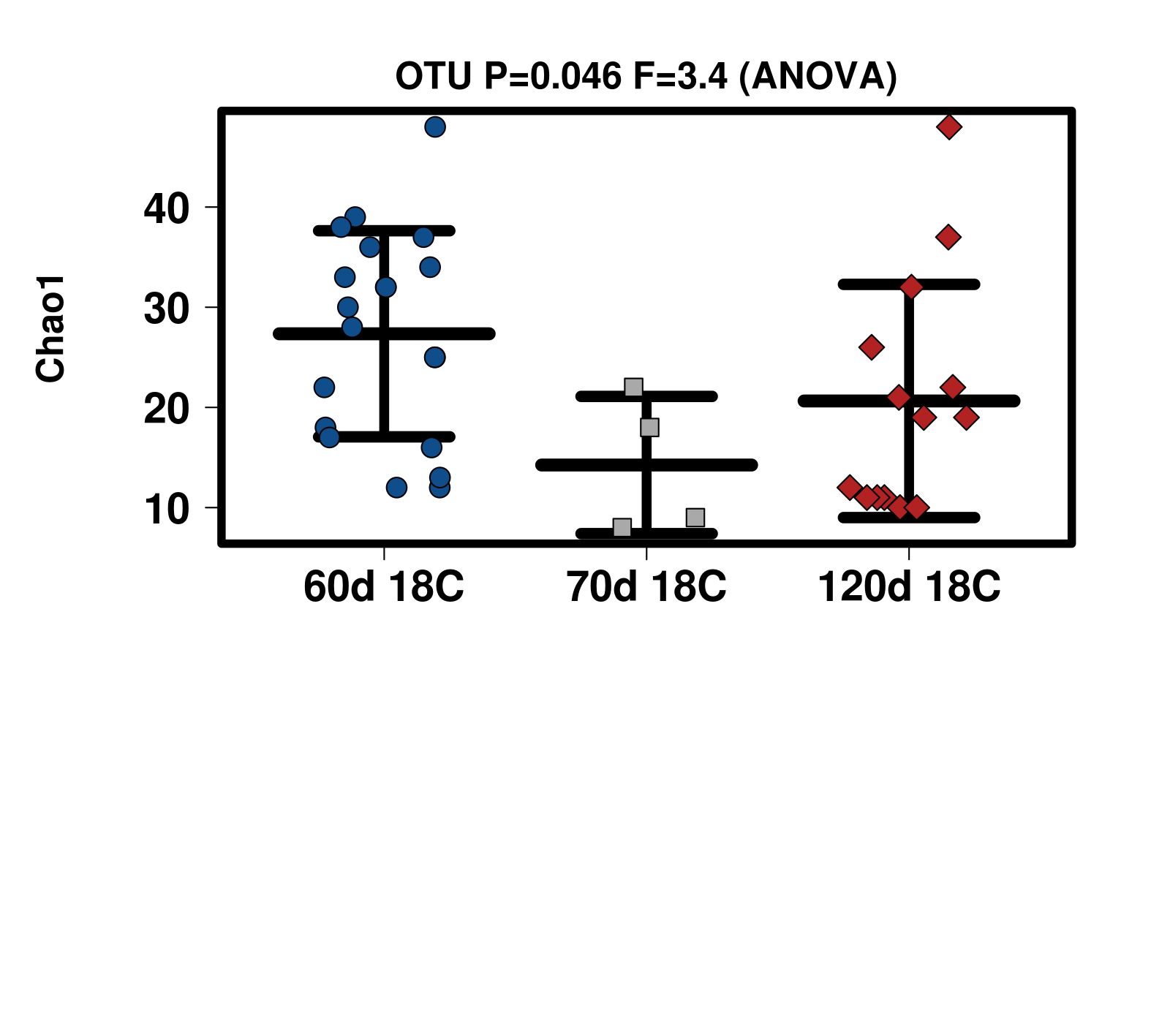


**Supplementary Figure 4.** Identification and quantification of mycotoxins produced by dry cured sausage *Penicillium* isolates.

**Supplementary Figure 5.** Mean populations of lactic flora and total aerobic mesophilic flora in meat of the *P. nalgiovense*-sprayed (A) and *P. nordicum-*sprayed (B) fermented dry sausages during ripening/drying processes (from D0 to D30) and subsequent storage (from D30 to D50).


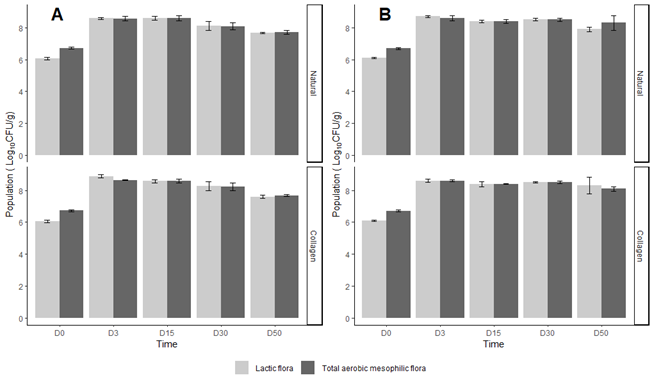

Supplement: Supplementary file 2 [file Data_Sheet_1.docx]
